# Functional Enhancement of Electrofusion-derived BRIN-BD11 Insulin-secreting Cells After Implantation into Diabetic Mice

**DOI:** 10.1155/EDR.2001.29

**Published:** 2001

**Authors:** Emma L. Davies, Yasser H. A. Abdel-Wahab, Peter R. Flatt, Clifford J. Bailey

**Affiliations:** 1 School of Life and Health Sciences Aston University Birmingham B4 7ET UK; 2 School of Biomedical Sciences University of Ulster Coleraine BT52 1SA UK

## Abstract

Electrofusion-derived BRIN-BD11 cells are glucosesensitive insulin-secreting cells which provide an archetypal bioengineered surrogate β-cell for insulin replacement therapy in diabetes mellitus, 5x10^6^ BRIN-BD11 cells were implanted intraperitoneally into severely hyperglycaemic (>24mmol/l) streptozotocin-induced insulin-treated diabetic athymic nude (*nu/nu*) mice. The implants reduced hyperglycaemia such that insulin injections were discontinued by 5–16 days (<17mmol/l) and normoglycaemia (<9mmol/l) was achieved by 7–20 days. Implanted cells were removed after 28 days and re-established in culture. After re-culture for 20 days, glucose-stimulated (16.7mmol/l) insulin release was enhanced by 121% (p<0.001) compared to non-implanted cells. Insulin responses to glucagon-like peptide-1 (10^−9^mol/l), cholecystokinin-8 (10^−8^ mol/l) and L-alanine (10 mmol/l) were increased by 32%, 31% and 68% respectively (p<0.05–0.01). Insulin content of the cells was 148% greater at 20 days after re-culture than before implantation (p<0.001), but basal insulin release (at 5.6 mmol/l glucose) was not changed. After re-culture for 40 days, insulin content declined to 68% of the content before implantation (p<0.01), although basal insulin release was unchanged. However, the insulin secretory responses to glucose, glucagonlike peptide-1, cholecystokinin-8 and L-alanine were decreased after 40 days of re-culture to 65%, 72%, 73% and 42% respectively of the values before implantation (p<0.05–0.01). The functional enhancement of electrofusion-derived surrogate β-cells that were re-cultured for 20 days after implantation and restoration of normoglycaemia indicates that the *in vivo* environment could greatly assist β-cell engineering approaches to therapy for diabetes.


*Int. Jnl. Experimental Diab. Res.*, Vol. 2, pp. 29–36
Reprints available directly from the publisher
Photocopying permitted by license only

© 2001 OPA (Overseas Publishers Association) N.V.
Published by license under
the Harwood Academic Publishers imprint,
part of Gordon and Breach Publishing,
a member of the Taylor & Francis Group.
Printed in the United States.

### Functional Enhancement of Electrofusion-derived BRIN-BD11 Insulin-secreting Cells After Implantation into Diabetic Mice

EMMA L. DAVIES[a], YASSER H. A. ABDEL-WAHAB[b], PETER R. FLATT[b] and CLIFFORD J. BAILEY[a,*]

[a]*School of Life and Health Sciences, Aston University, Birmingham B4 7ET, UK;*
[b]*School of Biomedical Sciences, University of Ulster, Coleraine BT52 1SA, UK*

*(Received 30 June 2000; In final form 19 October 2000)*

**Electrofusion-derived BRIN-BD11 cells are glucose-sensitive insulin-secreting cells which provide an archetypal bioengineered surrogate β-cell for insulin replacement therapy in diabetes mellitus. $5\times10^6$ BRIN-BD11 cells were implanted intraperitoneally into severely hyperglycaemic (>24mmol/l) streptozotocin-induced insulin-treated diabetic athymic nude (*nu/nu*) mice. The implants reduced hyperglycaemia such that insulin injections were discontinued by 5–16 days (<17mmol/l) and normoglycaemia (<9mmol/l) was achieved by 7–20 days. Implanted cells were removed after 28 days and re-established in culture. After re-culture for 20 days, glucose-stimulated (16.7mmol/l) insulin release was enhanced by 121% (p<0.001) compared to non-implanted cells. Insulin responses to glucagon-like peptide-1 ($10^{-9}$mol/l), cholecystokinin-8 ($10^{-8}$mol/l) and L-alanine (10mmol/l) were increased by 32%, 31% and 68% respectively (p<0.05–0.01). Insulin content of the cells was 148% greater at 20 days after re-culture than before implantation (p<0.001), but basal insulin release (at 5.6mmol/l glucose) was not changed. After re-culture for 40 days, insulin content declined to 68% of the content before implantation (p<0.01), although basal insulin release was unchanged. However, the insulin secretory responses to glucose, glucagon-like peptide-1, cholecystokinin-8 and L-alanine were decreased after 40 days of re-culture to 65%, 72%, 73% and 42% respectively of the values before implantation (p<0.05–0.01). The functional enhancement of electrofusion-derived surrogate β-cells that were re-cultured for 20 days after implantation and restoration of normoglycaemia indicates that the *in vivo* environment could greatly assist β-cell engineering approaches to therapy for diabetes.**

*Keywords*: BRIN-BD11 cells; Electrofusion; Insulin-secretion; Implantation; Diabetes

### INTRODUCTION

Cultured insulin-secreting cell lines offer a potential alternative to isolated pancreatic islets for implantation into patients with insulinopenic forms of diabetes mellitus.[2,8,18,20] Indeed, considerable effort has been devoted in the past five years to generation of new glucose-responsive islet cell lines and cellular engineering of existing insulin-secreting cells to confer attributes responsible for glucose sensitivity.[7,19,21,29] Use

*Corresponding author. Tel.: +44 (0) 121 359 3611, Fax: +44 (0) 121 359 0733, e-mail: c.j.bailey@aston.ac.uk of cultured cells for transplantation obviates the shortage of donor islets and difficulties of islet isolation and storage.[8,25] However, there are important unresolved issues concerning the implantation of insulin-secreting cell lines *in vivo*, including their containment, control of proliferation, immunological isolation, and responsiveness to glucose.[2,4,18,25]

Insulinoma-derived insulin-secreting cell lines generally show limited responsiveness to changes in extracellular glucose concentrations within the physiological range.[11,14,18,19,21] Electrofusion technology provides a method to produce immortal hybrid cells and this technology has recently been applied to the development of glucose-sensitive insulin-secreting cells.[18,19] BRIN-BD11 cells were derived by electrofusion of New England Deaconess Hospital (NEDH) rat pancreatic β-cells with the RINm5F cell line, which originated from an insulinoma in an NEDH rat.[17] BRIN-BD11 cells show increased insulin content compared with RINm5F cells. Additionally, and unlike RINm5F cells,[11] BRIN-BD11 cells show regulated insulin secretion in response to physiological concentrations of glucose and a wide range of other stimulators and inhibitors of pancreatic β-cell function.[6,17,18,19,26]

This study has examined the ability of electrofusion-derived BRIN-BD11 cells to reverse hyperglycaemia after implantation into severely insulinopenic streptozotocin (STZ)-diabetic immunotolerant athymic nude (*nu/nu*) mice. To evaluate whether the *in vivo* environment can assist β-cell engineering, the study has also assessed insulin content and insulin-secretory responsiveness of the surrogate β-cells retrieved after 28 days of implantation and re-culture *in vitro*.

### MATERIALS AND METHODS

### Chemicals and Animals

Chemicals of analytical grade were from Sigma Chemicals (Poole, UK) and BDH (Poole, UK). $^{125}$I-insulin was purchased from Amersham International (Amersham, UK). Human lentard insulin was from NovoNordisk (Copenhagen, Denmark) and glucose assay reagents were from Beckman (High Wycombe, UK). Tissue culture media, fetal calf serum, antibiotics and trypsin were from Gibco BRL (Paisley, UK) and plastic ware was from Sarstedt (Leicester, UK).

Adult male athymic nude (*nu/nu*) mice were obtained from Charles River (Margate, UK) and maintained in an air conditioned room at 22±2°C with a lighting schedule of 12h light (0800–2000h) and 12h dark. A standard pellet diet (Economy Rodent Diet, SDS, Great Waltham, UK) with tap water were supplied ad libitum. Mice were made diabetic by intraperitoneal (ip) injection of streptozotocin (200 mg/kg in 0.5mmol/l sodium citrate buffer, pH 4.5) following a 6h fast. Food was then withheld for a further 6h. Twice daily subcutaneous insulin injections (20U/kg human lentard) were started after 5–7 days when plasma glucose concentrations exceeded 20 mmol/l.

### BRIN-BD11 Cell Culture and *In Vitro* Studies

BRIN-BD11 cells were cultured in RPMI-1640 medium containing 11.1mmol/l glucose, 10% (v/v) foetal calf serum, 100IU/ml penicillin and 0.1mg/ml streptomycin, with 95% air and 5% $CO_2$ at 37°C. The production, culture and functional characterisation of this cell line are described in detail elsewhere.[17–19] Cells were maintained in 75cm$^3$ flasks and used at passages 15–36 after gently washing in 10ml Hank's buffered saline solution (HBSS), detaching with 0.025% (w/v) trypsin in normal saline containing 2g/l EDTA. For studies of insulin release and cellular insulin content, 2.5×10$^5$ cells were seeded per well in 1ml medium using 24-well multiplates, and cultured for 24h as above. Prior to acute tests, cells were preincubated for 40 min at 37°C in 1ml Krebs Ringer bicarbonate buffer (KRBB) containing 1.1mM glucose and 0.1% bovine serum albumin (BSA). Test incubations were then performed for 20min at 37°C in 1ml KRBB containing 0.1% BSA plus glucose and other stimulators of insulin release as indicated in the tables. Insulin content was measured by acid-ethanol extraction in 0.5ml of 1.5% (v/v) 0.7mol/l HCl, 75% (v/v) ethanol and 23.5% (v/v) water for 24h at 4°C. Test buffer and acid-ethanol extracts were stored at –20°C for subsequent insulin analysis.

### Implantation Studies

BRIN-BD11 cells, maintained in RPMI-1640 as above, and HepG2 cells (kindly donated by Professor Kevin Docherty, Aberdeen, UK) maintained in Dulbecco's modified Eagle's medium (DMEM) with Glutamax I supplemented with 10% foetal calf serum, 100IU/ml penicillin and 0.1mg/ml streptomycin, with 95% air and 5% $CO_2$ at 37°C were harvested by trypsinisation. The cell monolayer was gently washed with phosphate-buffered saline (PBS), then 1.5ml of 0.025% (w/v) trypsin in PBS containing 2g/l EDTA was added, the flask was incubated at 37°C until the cells detached, when 8.5ml of complete medium was added to stop trypsinisation. The cells were harvested by centrifugation at 1000 rpm using an MSE benchtop centrifuge and resuspended at a concentration of $5\times10^6$ in 200μl of complete medium. $5\times10^6$ cells in 200μl were injected ip into insulin-treated diabetic nude mice.

Blood samples were taken from the tail tip for determination of plasma glucose and on occasions plasma insulin.[10] On days 19–20 an oral glucose tolerance test (OGTT) was performed on overnight-fasted animals. The glucose dose was 2g/kg body weight, administered as a 40% w/v solution (*i.e.*, 5ml/kg) by oral gavage. Blood samples were obtained as above immediately before (time 0) and at 20, 40, 60 and 80min after administration of the glucose load. Animals were killed at 28 days, or earlier if hypoglycaemia supervened, and autopsied. Tumour-like aggregations were preserved in 10% formaldehyde in PBS and 3–5μm sections were stained for general morphology using haematoxylin and eosin, and for immunohistochemical visualisation of insulin using a guinea pig anti-insulin antibody with a DAKO Duet kit (DAKO, High Wycombe, UK).

To re-culture *ex-vivo* cells, tumour-like aggregations were aseptically excised and cut into small pieces in a small volume of complete medium and disaggregated using a 21 gauge needle. The resulting cell suspension was cultured overnight, using the standard conditions for BRIN-BD11 cells, after 24h the cells were washed with PBS to remove any remaining aggregates and fresh medium was added.

### Analyses

Plasma glucose was measured by an automated glucose oxidase procedure[28] and insulin was measured by dextran-charcoal radioimmunoassay[10] using a fully cross-reacting antibody and rat insulin as standard. Groups of data were compared using the Student's unpaired t-test. Differences were considered to be significant if $p<0.05$.

### RESULTS

### Implantation Studies

Induction of diabetes with a high dose of streptozotocin (200mg/kg, ip) produced severe hyperglycaemia in the nude (*nu/nu*) mice. To avoid mortality, all mice were treated with twice daily injections of insulin (20U/kg human lentard, sc). Plasma glucose concentrations, which were measured 18h after the last insulin injection, were consistently in the range 21–30mmol/l (Fig. 1), compared with the values of 6–9mmol/l in normal non-diabetic untreated *nu/nu* mice.

After 2 weeks of severe but stable insulin-treated diabetes, mice received an intraperitoneal implant of either $5\times10^6$ BRIN-BD11 cells, or an intraperitoneal implant of $5\times10^6$ HepG2 cells a s a control. As shown in Figure 1 the non-insulin-secreting HepG2 cells did not affect plasma glucose control, and insulin injections were continued. However, in mice receiving the implant of insulin-secreting BRIN-BD11 cells, plasma glucose concentrations were reduced at varying rates. When the glucose concentration

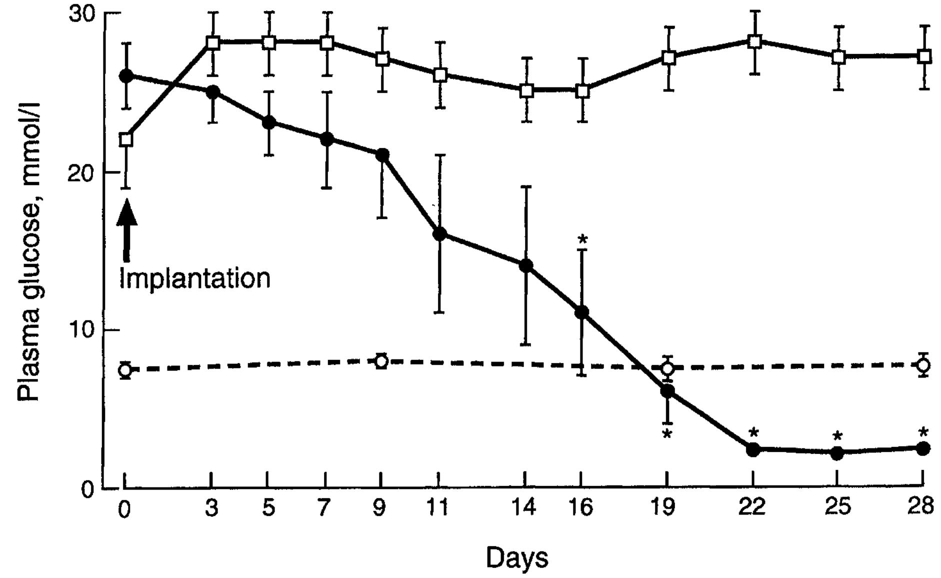

FIGURE 1 Plasma glucose concentrations of untreated non-diabetic nude (*nu/nu*) mice (O ---- O) and streptozotocin diabetic nude (*nu/nu*) mice implanted intraperitoneally on day 0 with $5\times10^6$ insulin-secreting BRIN-BD11 cells (● —— ●) or $5\times10^6$ non-insulin-secreting HepG2 cells (□ —— □). Values are mean±sem of 5 (untreated and HepG2) or 6 (BRIN-BD11) mice. *p<0.05 *versus* mice treated with HepG2 cells.

fell below 20mmol/l the insulin dosage was reduced by half, and when glycaemia fell below 17mmol/l insulin injections were discontinued. Insulin injections were discontinued in all mice implanted with the BRIN-BD11 cells (5–16 days after implantation), and all of these mice achieved normal plasma glucose concentrations (<9mmol/l) by 7–20 days after implantation. An oral glucose tolerance test (2g/kg) conducted on overnight fasted mice 19–20 days after cell implantation showed lower plasma glucose concentrations than normal non-diabetic untreated *nu/nu* mice (Fig. 2).

Plasma glucose concentrations fell below 2 mmol/l by 11–22 days after implantation of BRIN-BD11 cells (Fig. 1), and the study was terminated at 28 days. At this time, plasma insulin concentrations ranged from 1.2 to 14.0ng/ml (mean±sem, 7.6±3.1ng/ml) in the mice implanted with the BRIN-BD11 cells. In untreated control mice and mice implanted with HepG2 cells basal plasma insulin concentrations were <1 ng/ml.

Autopsy showed that ip implants of BRIN-BD11 cells produced multiple well-vascularised aggregations of cells attached to the peritoneal wall, diaphragm, mesentery and occasionally attached to the liver and kidney. The aggregates were typically 1–5mm in diameter. They comprised a thick outer mantle rich in healthy insulin-positive staining cells with areas of necrosis dispersed towards the core. The many small aggregates in each recipient precluded accurate quantitation of their overall weight or total number of insulin-positive staining cells. HepG2 implants produced similar aggregates but were devoid of insulin-positive staining cells and showed fewer areas of necrosis.

In a separate study, three insulin-treated STZ-diabetic *nu/nu* mice received subcutaneous (sc) implants of $5\times10^6$ BRIN-BD11 cells in the suprascapular region. Following the same protocol as for the ip implants, the sc implanted mice showed a reduction in hyperglycaemia. Insulin injections were discontinued at 9–15 days and the study was terminated at 24 days

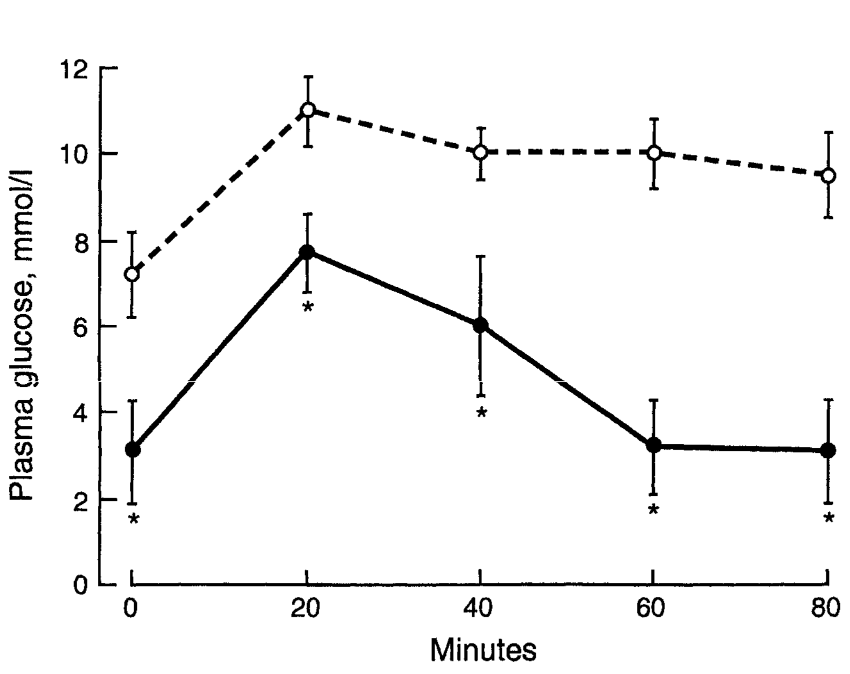

FIGURE 2 Effect of glucose (2g/kg body weight given orally at 0min) on plasma glucose concentrations of untreated non-diabetic nude (*nu/nu*) mice (O --- O) and streptozotocin diabetic nude (*nu/nu*) mice at 19–20 days after intraperitoneal implantation of $5\times10^6$ insulin-secreting BRIN-BD11 cells (● —— ●). Values are mean±sem of 5 (untreated) or 6 (BRIN-BD11) mice. *P<0.05 *versus* untreated mice.

when glucose concentrations had fallen below 2mmol/l in one mouse. Multiple aggregates of insulin-positive staining cells were dispersed in the suprascapular region at autopsy.

### Insulin Secretion *In Vitro*

Before implantation, the BRIN-BD11 cells showed a 2.5 fold increase (p<0.001) of insulin release when the glucose concentration was raised from 5.6 to 16.7mmol/l (Tab. I). L-Alanine (10mmol/l) produced a marked increase (9.2 fold, p<0.001) of insulin release, while glucagon-like peptide-1 (7–36 amide) (tGLP-1; $10^{-9}$mol/l) and cholycystokinin-8 (CCK-8; $10^{-8}$mol/l) increased insulin release 2.7 fold and 2.1 fold respectively (p<0.001).

After implantation in *nu/nu* mice for 28 days,

TABLE I Insulin secretion by BRIN-BD11 cells during 20min incubations *in vitro* before and after establishment in re-culture following 28 days implantation in diabetic nude mice

| Test agent (M) | Glucose (mM) | Before implantation | Insulin secretion ng/$10^6$ cells/20 min — Days of re-culture: 9 | 20 | 40 |
|---|---|---|---|---|---|
| None | 5.6 | 0.9 ± 0.1 | 0.8 ± 0.1 | 0.9 ± 0.1 | 0.9 ± 0.1 |
| None | 16.7 | 2.3 ± 0.2$^{b}$ | 5.1 ± 0.7$^{bd}$ | 5.1 ± 0.5$^{be}$ | 1.5 ± 0.1$^{ad}$ |
| L-alanine ($10^{-2}$) | 5.6 | 8.3 ± 1.5$^{b}$ | 11.4 ± 0.6$^{bc}$ | 13.9 ± 0.9$^{bc}$ | 3.5 ± 0.3$^{bc}$ |
| tGLP-1 ($10^{-9}$) | 5.6 | 2.5 ± 0.3$^{b}$ | 3.8 ± 0.2$^{bd}$ | 3.3 ± 0.2$^{b}$ | 1.8 ± 0.1$^{bc}$ |
| CCK-8 ($10^{-8}$) | 5.6 | 1.9 ± 0.1$^{b}$ | 2.7 ± 0.1$^{be}$ | 2.5 ± 0.1$^{bd}$ | 1.4 ± 0.1$^{bc}$ |

Values are mean±sem, n = 6; $^{a}$p<0.01, $^{b}$p<0.001 compared with 5.6mM glucose at the same time; $^{c}$p<0.05, $^{d}$p<0.01, $^{e}$p<0.001 compared with the same test agent before implantation. tGLP-1, glucagon-like peptide-1 (7–36 amide); CCK-8, cholecystokinin-8.

the aggregations of implanted cells were excised and the cells were re-established in culture for up to 40 days. At 9 and 20 days of re-culture the insulin responses to a rise in the glucose concentration from 5.6mmol/l to 16.7mmol/l glucose were 6.3 and 5.6 fold respectively ($p<0.001$). These responses were significantly greater ($p<0.01$ and $p<0.001$) than the 2.5 fold increase achieved before implantation. The insulin responses to 10mmol/l L-alanine, $10^{-9}$mol/l tGLP-1 and $10^{-8}$mol/l CCK-8 were also significantly greater than before implantation (Tab. I). The insulin content per cell at 9 and 20 days after re-culture was 1.6–2.4 fold higher ($p<0.05$ –0.001) than before implantation (Fig. 3).

At 40 days of re-culture the insulin responses to glucose, L-alanine, tGLP-1 and CCK-8 declined to below the values observed before implantation (Tab. I), and the insulin content had fallen to 32%

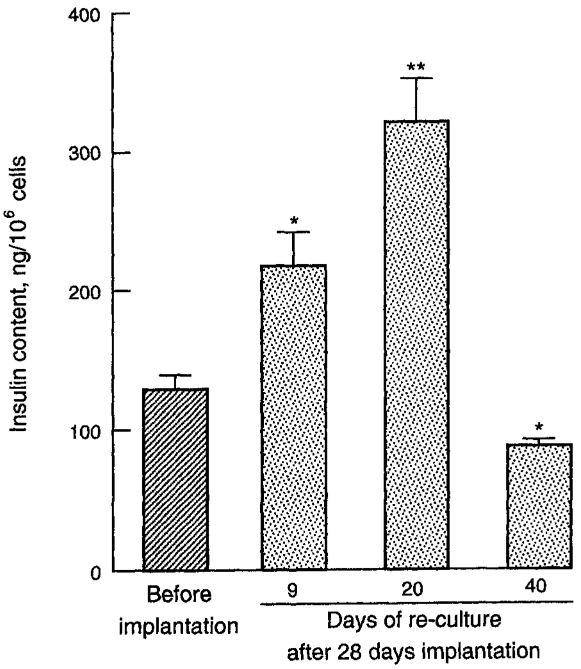

FIGURE 3 Insulin content of BRIN-BD11 cells before intraperitoneal implantation in streptozotocin diabetic nude (*nu/nu*) mice, and at 9, 20 and 40 days of re-culture after implantation for 28 days. Values are mean±sem, n=6. *$p<0.05$, **$p<0.001$ *versus* before implantation.

below ($p<0.01$) that before implantation (Fig. 3). Basal insulin secretion at 5.6mmol/l glucose was not affected by *in vivo* implantation or the period of subsequent re-culture (Tab. I). Non-implanted cells maintained in culture for the same number of passages showed no significant changes in insulin secretion or content.[17]

### DISCUSSION

BRIN-BD11 is a unique cell line produced by the application of electrofusion technology to an insulinoma-derived insulin-secreting cell line.[17] Although the insulin content of BRIN-BD11 cells is only modestly greater than the parent RINm5F cell line, the insulin secretory responses of BRIN-BD11 cells show similarities to normal β-cells when exposed to a wide range of established stimulators and inhibitors of pancreatic β-cell function, including nutrients, hormones, neurotransmitters and drugs.[17–19] Consistent with these early studies, BRIN-BD11 cells used in the present experiments showed insulin secretory responses to glucose, L-alanine, tGLP-1 and CCK-8.[1,17,22]

The therapeutic potential of electrofusion-derived insulin-secreting cells has been demonstrated herein by the lowering of plasma glucose concentrations and elimination of the need for exogenous insulin injections after intraperitoneal implantation of BRIN-BD11 cells in severely diabetic insulin-treated nude mice. These observations parallel studies using intact islets, dispersed islet cells or immunoprotected MIN-6-cells,[4,13,15,16,23] from which it is evident that the number of implanted cells required to reverse hyperglycaemia is greater than the complement of β-cells in a normal pancreas.

The well vascularised tumour-like aggregations of rat-derived BRIN-BD11 cells after 28 days of implantation in diabetic nude mice showed no signs of immune rejection, consistent with the immunocompromised status of the host.[24] Resection of the cells after 28 days of implantation and re-establishment in culture for 9 and 20 days significantly enhanced insulin content and increased insulin secretory responsiveness to glucose, L-alanine, tGLP-1 and CCK-8 compared with cells which were not previously implanted. Interestingly basal insulin secretion was unchanged, suggesting a functional enhancement of β-cell stimulus-secretion coupling pathways rather than an explanation based simply upon enhanced cellular insulin content. The mechanisms responsible for these changes in secretory function require further investigation, but they seem likely to include *in vivo* enhancement in the expression of functional genes involved in glucose-sensing and insulin exocytosis. Candidates include Glut-2, glucokinase and other components of the signal transduction pathways used by the secretagogues tested, possibly including $K^+$-ATP channels, voltage-dependent $Ca^{2+}$ channels, cyclic AMP, protein kinase A, phospholipase C, inositol 1,4,5 trisphosphate, diacylglycerol and protein kinase C.[3,9,12] Additional or alternative possibilities include alterations in the pathways of cellular maturation.

Although follow-up experiments indicate that the functional enhancement persisted to 20 days in re-culture, the attributes gained *in vivo* were totally lost by 40 days of re-culture. Indeed, by 40 days the functional capacity of the resected cells was inferior to cells maintained in parallel *in vitro*. The reasons for such a decline are unclear but could stem from differences in the rate of proliferation and degree of differentiation during *in vivo* maintenance. These observations clearly support the idea that *in vivo* factors exert a significant en-hancement of functional gene expression[27] that persists for several days in BRIN-BD11 cells after re-section and reculture. Recent studies suggest that tGLP-1 might constitute one such factor.[30,31] However, the present experimental system might provide an opportunity to identify the full spectrum of factors and target genes involved.

Despite unchanged basal insulin secretion and improved glucose-regulated insulin secretion of BRIN-BD11 cells during *in vitro* studies at 9 and 20 days after re-section, plasma glucose concentrations of the diabetic nude mice declined to hypoglycaemic levels by 11–22 days after implantation. This indicates that the capacity of implanted cells to limit insulin secretion in the face of low plasma glucose was insufficient to compensate for the increasing β-cell mass. Strategies are being developed in other laboratories to offset this problem, for example by incorporating an antibiotic-sensitive inducer gene into surrogate β-cells to limit cell growth,[5,8] and by the use of encapsulation and prosthetic implantation devices.[4,13,16,23]

In conclusion, the present study has demonstrated the capacity of electrofusion-derived insulin-secreting cells to reverse the hyperglycaemia of severely diabetic nude mice. Functional enhancement of these cells by the *in vivo* environment indicates that the true utility of engineered surrogate β-cells for transplantation can only be properly assessed *in vivo*. Further, it seems likely that if a surrogate β-cell has been endowed with appropriate genes from parental pancreatic β-cells, the expression of these genes, and hence the insulin secretory function of the cells, can be very usefully enhanced by the *in vivo* environment. Overall, these observations illustrate the potential value of multiple gene transfer by electrofusion to generate limitless numbers of surrogate β-cells for possible therapy of diabetes.

### *References*

[1] Abdel-Wahab, Y. H. A., O'Harte, F. P. M., Mooney, M., Conlon, J. M. and Flatt, P. R. (1999). Glycation of CCK-8 abolishes its insulinotropic action on clonal pancreatic B-cells. *Biochim. Biophys. Acta*, **1452**, 60–67.

[2] Bailey, C. J., Davies, E. L. and Docherty, K. (1999). Prospects of insulin delivery by ex-vivo somatic cell gene therapy. *J. Mol. Med.*, **77**, 244–249.

[3] Berggren, P. O., Rorsman, P., Efendic, S., Ostenson, C. G., Flatt, P. R., Nilsson, T., Arkhammar, P. and Juntti-Berggren, L., Mechanisms of action of enteroinsular hormones, islet peptides and neural input on the insulin secretory process. In: Flatt, P. R. Ed., *Nutrient Regulation of Insulin Secretion*. London: Portland Press, 1992, pp. 289–318.

[4] Berney, Y. and Ricordi, C. (1999). Islet transplantation. *Cell Transplant.*, **8**, 461–464.

[5] Brownlee, M. (1997). Generation of nonimmune islet cells using genetic engineering. *J. Mol. Med.*, **77**, 230–234.

[6] Chapman, J. C., McClenaghan, N. H., Cosgrove, K., Ammala, C., Flatt, P. R. and Dunne, M. J. (1999). Regulation of ATP-sensitive channels and insulin release from a B-cell line, BRIN-BD11, generated by electrofusion. *Diabetes*, **48**, 2349–2357.
[7] Efrat, S. (1996). Genetic engineering of B-cells for cell therapy of diabetes: cell growth, function and immunogenicity. *Diabetes Rev.*, **4**, 224–234.
[8] Efrat, S. (1998). Cell-based therapy for insulin-dependent diabetes mellitus. *Eur. J. Endocrinol.*, **138**, 129–133.
[9] Flatt, P. R. Ed., *Nutrient Regulation of Insulin Secretion*. London: Portland Press, 1992.
[10] Flatt, P. R. and Bailey, C. J. (1981). Abnormal plasma glucose and insulin responses in heterozygous lean (ob/+) mice. *Diabetologia*, **20**, 573–577.
[11] Flatt, P. R., Bailey, C. J., Berggren, P. O., Herberg, L. and Swanston-Flatt, S. K., Defective insulin secretion in diabetes and insulinoma. In: Flatt, P. R. Ed., *Nutrient Regulation of Insulin Secretion*. London: Portland Press, 1992, pp. 341–386.
[12] Flatt, P. R. and Lenzen, S. (Eds.), *Frontiers of Insulin Secretion and Pancreatic B-cell Function*. London: Smith-Gordon, 1994.
[13] Hayashi, H., Inoue, K., Aung, T., Tun, T., Wang, W. J., Gu, Y. J., Shinohara, S., Echigo, Y., Kaji, H., Kato, M., Setoyama, H., Kawakami, Y., Imamura, M., Morikawa, N., Iwata, H., Ikada, Y. and Miyazaki, J. (1996). Prolongation of survival of a xenografted bioartificial pancreas with amesh-reinforced polyvinyl alcohol hydrogel bag employing a B-cell line (MIN6). *Transplant. Proc.*, **28**, 1097–1098.
[14] Hohmeier, H. E., Beltrandel-Rio, H., Clark, S. A., Henkel-Rieger, R., Normington, K. and Newgard, C. B. (1997). Regulation of insulin secretion from novel engineered insulinoma cell lines. *Diabetes*, **46**, 968–977.
[15] Inada, S., Kaneko, S., Suzuki, K., Miyazaki, J., Asakura, H. and Fujiwara, M. (1996). Rectification of diabetic state in C57BL/KsJ-db/db mice by the implantation of pancreatic beta cell line MIN6. *Diabetes Res. Clin. Pract.*, **32**, 125–133.
[16] Kawakami, Y., Inoue, K., Hayashi, H., Wang, W., Setoyama, H., Gu, Y. J., Imamura, M., Iwata, H., Ikada, Y., Nozawa, M. and Miyazaki, J. (1997). Subcutaneous xenotransplantation of hybrid artificial pancreas encapsulating pancreatic B cell line (MIN6): functional and histological study. *Cell Transplant.*, **6**, 541–545.
[17] McClenaghan, N. H., Barnett, C. R., Ah-Sing, E., Abdel-Wahab, Y. H. A., O'Harte, F. P. M., Yoon, T.-W., Swanston-Flatt, S. K. and Flatt, P. R. (1996). Characterization of a novel glucose-responsive insulin-secreting cell line, BRIN-BD11, produced by electrofusion. *Diabetes*, **45**, 1132–1140.
[18] McClenaghan, N. H. and Flatt, P. R. (1999). Engineering cultured insulin-secreting pancreatic B-cell lines. *J. Molec. Med.*, **77**, 235–243.
[19] McClenaghan, N. H. and Flatt, P. R. (1999). Physiological and pharmacological regulation of insulin release– insights offered through exploitation of insulin-secreting cell lines. *Diabetes, Obesity and Metab.*, **1**, 137–150.
[20] Newgard, C. B. (1994). Cellular engineering and gene therapy strategies for insulin replacement in diabetes. *Diabetes*, **43**, 341–350.
[21] Newgard, C. B., Clark, S., BeltrandelRio, H., Hohmeier, H. E., Quaade, C. and Normington, K. (1997). Engineered cell lines for insulin replacement in diabetes: Current status and future prospects. *Diabetologia*, **40**, S42–S47.
[22] O'Harte, F. P. M., Abdel-Wahab, Y. H. A., Conlon, J. M. and Flatt, P. R. (1998). Glycation of glucagon-like peptide-1 (7-36) amide: Characterisation and impaired action on rat insulin secreting cells. *Diabetologia*, **41**, 1187–1193.
[23] Ohgawara, H., Miyazaki, J., Nakagawa, Y., Sato, S., Karibe, S. and Akaike, T. (1996). Xenoimplantation using a diffusion chamber with a B-cell line (MIN6) as a bioartificial endocrine pancreas (Bio-AEP). *Cell Transplant.*, **5**, S71–S73.
[24] Pelleitier, M. and Montplaisir, S. (1975). The nude mouse: a model of deficient T-cell function. *Methods Achiev. Exp. Pathol.*, **7**, 149–66.
[25] Ricordi, C. (1996). Human islet cell transplantation: new perspectives for an old challenge. *Diabetes Rev.*, **4**, 356–369.
[26] Salgado, A. P., Santos, R. M., Pereira, F. C., Fernandes, A. P., Tome, A. R., Flatt, P. R., Ranasamy, R. and Rosario, L. M., Glucose signalling in clonal insulin-secreting BRIN-BD11 cells: Role of Cytosolic Ca2+ and redox state. In: Thierry, J. P. Ed., *Molecular Mechanisms of Transcellular Signalling*, Amsterdam , IOS Press, 1999, pp. 212–220.
[27] Sander, M. and German, M. S. (1997). The beta cell transcription factors and the development of the pancreas. *J. Mol. Med.*, **75**, 327–340.
[28] Stevens, J. F. (1971). Determination of glucose by automatic analyser. *Clin. Chem.*, **32**, 9199–9201.
[29] Tiedge, M., Elsner, M., McClenaghan, N. H., Hedrich, H.-J., Grube, D., Klempnauer, J. and Lenzen, S. (2000). Engineering of a glucose-responsive cell for insulin replacement therapy of experimental insulin-dependent diabetes. *Human Gene Ther.*, **11**, 403–414.
[30] Xu, G., Stoffers, D. A., Habener, J. F. and Bonner-Weir, S. (1999). Exendin-4 stimulates both B-cell replication and neogenesis, resulting in increased B-cell mass and improved glucose tolerance in diabetic rats. *Diabetes*, **48**, 2270–2276.
[31] Zhou, J., Wang, X., Pineyro, M. A. and Egan, J. M. (1999). Glucagon-like peptide 1 and exendin-4 convert pancreatic AR42J cells into glucagon- and insulin-producing cells. *Diabetes*, **48**, 2358–2366.

